# Comprehensive Analysis of the Immune Infiltrates of Pyroptosis in Kidney Renal Clear Cell Carcinoma

**DOI:** 10.3389/fonc.2021.716854

**Published:** 2021-09-09

**Authors:** Zhuolun Sun, Changying Jing, Xudong Guo, Mingxiao Zhang, Feng Kong, Zhenqing Wang, Shaobo Jiang, Hanbo Wang

**Affiliations:** ^1^Department of Urology, Third Affiliated Hospital, Sun Yat-sen University, Guangzhou, China; ^2^School of Medicine, Technical University of Munich, Munich, Germany; ^3^Department of Urology, Shandong Provincial Hospital Affiliated to Shandong First Medical University, Jinan, China; ^4^Department of Urology, The First Affiliated Hospital of Sun Yat-sen University, Guangzhou, China

**Keywords:** pyroptosis, kidney renal clear cell carcinoma, tumor microenvironment, survival analysis, prognostic model

## Abstract

Kidney renal clear cell carcinoma (KIRC) has long been identified as a highly immune-infiltrated tumor. However, the underlying role of pyroptosis in the tumor microenvironment (TME) of KIRC remains poorly described. Herein, we systematically analyzed the prognostic value, role in the TME, response to ICIs, and drug sensitivity of pyroptosis-related genes (PRGs) in KIRC patients based on The Cancer Genome Atlas (TCGA) database. Cluster 2, by consensus clustering for 24 PRGs, presented a poor prognosis, likely because malignancy-related hallmarks were remarkably enriched. Additionally, we constructed a prognostic prediction model that discriminated well between high- and low-risk patients and was further confirmed in external E-MTAB-1980 cohort and HSP cohort. By further analyzing the TME based on the risk model, higher immune cell infiltration and lower tumor purity were found in the high-risk group, which presented a poor prognosis. Patients with high risk scores also exhibited higher ICI expression, indicating that these patients may be more prone to profit from ICIs. The sensitivity to anticancer drugs that correlated with model-related genes was also identified. Collectively, the pyroptosis-related prognosis risk model may improve prognostic information and provide directions for current research investigations on immunotherapeutic strategies for KIRC patients.

## Introduction

Renal cell carcinoma (RCC) is one of the most prevalent urologic malignancies worldwide, with an estimated annual incidence of 14,000 cancer-related deaths in the United States (1). Approximately 30% of patients harbor distant metastases at the time of diagnosis (2). Patients with metastatic RCC (mRCC) present a poor prognosis and have a 10% 5-year survival rate, in contrast to that of non-RCC with an estimated rate of over 55% (3). Kidney renal clear cell carcinoma (KIRC) is the most frequent histological type and is responsible for approximately 70% of all cases of RCC in adults (4). Surgical resection remains the primary treatment modality in most patients with KIRC; however, 30%–40% of patients with localized disease develop metastatic recurrence during follow-up following surgical resection (2). The role of immune infiltrations in cancer development has become the focus of much research. Numerous studies have demonstrated that the different immune cell infiltrates present in the tumor are closely related to the clinical outcomes in some human malignancies (5). KIRC has long been identified and proven to be a highly infiltrated tumor in genomic studies and clinical settings (6). It has been estimated that up to 1% of spontaneous KIRC regression is accompanied by signs of immune mediation (7). Historically, KIRC is one of the first malignant tumors to respond to immunotherapy and remains one of the most sensitive (8). The recent development of cancer immunotherapies such as immune checkpoint inhibitors (ICIs) has revolutionized traditional cancer therapy because of its safety and efficacy (9). However, the response of KIRC to immunotherapy has been unsatisfactory, as expected, and effective disease control and therapeutic strategies are required for further improvements (10). The tumor microenvironment (TME) represents the primary site of continuous interaction between neoplastic and immune system cells, and its various components are associated with tumor progression and therapeutic outcomes (11, 12). Additionally, multiple cytokines and various immunosuppressive cells are involved in tumor immune escape in the KIRC microenvironment (13). Thus, understanding the regulatory mechanism of the TME is critical to identify efficient prognostic biomarkers and optimize individualized immunotherapy regimens against cancer.

The inflammasome is a large cytosolic multiprotein complex that forms a key component of the innate immune system (14). Pyroptosis, recognized as a highly specific inflammatory programmed cell death, is triggered by caspase-1 and -11 (also known as caspase-4 or -5 in humans) in the canonical and noncanonical pathways, respectively (15). Pyroptosis results in cell and organelle swelling, membrane lysis, DNA cleavage, and the release of intracellular proinflammatory contents such as interleukin-1β (IL-1β), which induces local or systemic inflammatory effects (16). Recently, pyroptosis was proven to be closely related to various human diseases, particularly malignant tumors. Pyroptosis plays a dual role during tumor progression (17). During pyroptosis, the various inflammatory mediators derived from the activation of signaling pathways affect tumorigenesis. For example, as an essential part of pyroptosis, NLRP1 mediates caspase-1-dependent secretion of IL-1β and IL-18 cytokines, which promote skin cancer (18). Miguchi et al. confirmed that TGFBR2 mutation upregulates the expression of GSDMC, facilitating colorectal tumor cell proliferation and tumorigenesis (19). Additionally, as a type of death, pyroptosis suppresses tumor development and progression. Wang et al. reported that the downregulation of GSDMD accelerated the S/G_2_ cell transition to accelerate gastric cancer cell proliferation by regulating cell cycle-related proteins (20).

Currently, most studies have focused primarily on the intrinsic oncogenic pathways of malignant tumors, and the function and underlying mechanism of pyroptosis in the TME remain unelucidated. Erkes et al. demonstrated that an intact immune system, particularly CD4+ and CD8+ T cells, is required for the efficacy of BRAF inhibitors and MEK inhibitors (BRAFi + MEKi) in melanoma (21). BRAFi + MEKi trigger the activation of caspase-3, causing the cleavage of GSDME, which is a hallmark of pyroptosis of tumor cells and is essential for T-cell activation and tumor regression. The secondary pyroptosis mediated by the caspase 3-dependent cleavage of GSDME could be an indispensable intermediary of immune-driven treatment responsiveness, revealing a potential therapeutic target in enhancing immunotherapy efficacy. Accordingly, pyroptosis-related genes (PRGs) involved in regulating the tumor immune response might be recognized as potential targets in potentiating the clinical activity of immunotherapies. Nevertheless, a complete understanding of pyroptosis in KIRC, including the interactions between pyroptosis and the TME, remains limited.

In the current work, the constructed clustering subtypes and pyroptosis-related risk model were essential for improving clinical risk stratification to make management decisions and predict prognosis for patients with KIRC. Additionally, we thoroughly analyzed the prognostic value, role in the TME, response to ICIs, and drug sensitivity of PRGs in KIRC patients based on the pyroptosis-related prognosis model to further study the effects of pyroptosis on the TME. We performed the present study to provide a novel perspective and a more detailed understanding of the immune infiltrates of pyroptosis and identify reliable prognostic predictors for KIRC patients.

## Materials and Methods

### Data Source

RNA sequencing transcriptome data harmonized to the fragments per kilobase million (FPKM) of 539 KIRC samples and 72 normal kidney tissues were downloaded from the TCGA database (https://tcga-data.nci.nih.gov/tcga/). The corresponding clinical characteristics, including age, gender, grade, AJCC stage, TNM stage, and survival status, were also extracted from TCGA. Patients with simultaneously available mRNA expression profiles and survival times (OS and DFS) > 0 days were enrolled in the study. In total, 525 patients were randomly split into a training cohort (60%; n = 317) and a testing cohort (40%; n = 208) *via* a 10‐fold cross‐validation method using the R package “caret”. The training cohort was used to construct the prognostic risk model, and the testing cohort and entire cohort were used to verify the predictive reliability and accuracy of the model. Additionally, the E-MTAB-1980 cohort downloaded from the ArrayExpress database (https://www.ebi.ac.uk/arrayexpress/) and Shandong Provincial Hospital (HSP) cohort were used as the external validation cohorts. The clinical characteristics of these patients are shown in **Table 1**.

**Table 1:** Characteristics of all patients included in this study.

Variable	Training cohort (n = 317)	Testing cohort (n = 208)	Entire cohort (n = 525)
	Number (%)	Number (%)	Number (%)
**Age**			
≤60	158(49.84)	106(50.96)	264(50.29)
>60	159(50.16)	102(49.04)	261(49.71)
**Gender**			
Female	109(34.38)	73(35.1)	182(34.67)
Male	208(65.62)	135(64.9)	343(65.33)
**Grade**			
G1	8(2.52)	5(2.40)	13(2.48)
G2	131(41.32)	95(46.67)	226(43.05)
G3	127(40.06)	77(37.02)	204(38.86)
G4	47(14.83)	27(12.98)	74(14.10)
unknow	4(1.26)	4(1.92)	8(1.52)
**AJCC stage**			
I	147(46.37)	114(71.28)	26149.71)
II	42(13.25)	14(6.73)	56(10.67)
III	75(23.66)	48(23.08)	123(23.43)
IV	52(16.40)	30(14.42)	82(15.62)
unknow	1(0.32)	2(0.96)	3(0.57)
**T stage**			
T1	150(47.32)	117(56.25)	267(50.86)
T2	49(15.46)	19(9.13)	68(12.95)
T3	111(35.02)	68(23.69)	179(34.1)
T4	7(2.21)	4(1.92)	11(2.10)
**N stage**			
N0	138(43.53)	99(47.6)	237(45.14)
N1-3	11(3.47)	5(2.4)	16(3.05)
unknow	168(53)	104(50)	272(51.81)
**M stage**			
M0	252(79.5)	165(79.33)	417(79.43)
M1	49(15.46)	29(13.94)	78(14.86)
unknow	16(5.05)	14(3.76)	30(5.71)

**Table:** 

Variable	E-MTAB-1980 cohort (n = 101)	HSP cohort (n = 186)
	Number (%)	Number (%)
**Age**		
≤60	44(41.90)	132(70.96)
>60	57(58.10)	54(29.04)
**Gender**		
Female	24(23.76)	121(65.05)
Male	77(76.24)	55(34.95)
**Grade**		
G1	13(12.87)	44(23.66)
G2	59(58.41)	102(54.84)
G3	22(21.78)	28(15.05)
G4	5(4.96)	12(6.45)
unknow	0(0.00)	0(0.00)
**AJCC stage**		
I	66(64.35)	102(54.54)
II	10(9.90)	36(19.36)
III	13(12.87)	24(12.90)
IV	12(11.88)	24(12.90)
unknow	0(0.00)	0(0.00)
**T stage**		
T1	68(67.33)	110(59.14)
T2	11(10.89)	42(22.58)
T3	21(20.79)	25(13.44)
T4	1(0.99)	9(4.84)
**N stage**		
N0	11(90.10)	176(94.62)
N1-3	10(9.90)	10(5.38)
unknow	0(0.00)	0(0.00)
**M stage**		
M0	94(93.07)	171(91.94)
M1	7(6.93)	15(8.06)
unknow	0(0.00)	0(0.00)

Next, 24 PRGs were retrieved from the previously published literature (22–24). The “limma” package was used to analyze differentially expressed PRGs between tumor tissues and adjacent normal pairs from TCGA.

### Consensus Clustering Analysis of PRGs

To investigate the biological characteristics of PRGs in KIRC patients, we classified the patients into different subtypes using the “ConsensusClusterPlus” package with a resampling rate of 80% and 50 iterations. PCA was performed to detect differences in gene expression patterns in distinct KIRC subtypes. The differentially expressed genes in different subtypes were subjected to biological process term GO functional annotation. To illustrate the functions associated with different subtypes of KIRC, GSEA was performed using the Hallmark gene set “h.all.v7.2.symbols.gmt” from the MSigDB database (http://www.broadinstitute.org/gsea) as previously described (25). GSEA significance was determined as a false discovery rate (FDR) ≤ 0.25 and nominal *p* ≤ 0.05.

### Construction and Evaluation of the Pyroptosis-Related Prognostic Risk Model

Univariate Cox proportional hazards regression analysis was used to assess the prognostic implication of every differentially expressed PRG, and then the features with a p value < 0.05 in the training cohort were defined as prognosis-related factors. Next, LASSO Cox regression analysis was performed to screen out the optimal gene combination to construct the risk model. The optimal values of the penalty parameter λ were finally determined by 10-fold cross-validation to construct an optimal LASSO regression model. The coefficient calculated by LASSO regression and gene expression level were applied to obtain the risk score formula as follows: Risk score = (expr_gene1_ × Coef_gene1_) + (expr_gene2_ × Coef_gene2_) + … + (expr_genen_ × Coef_genen_). Every KIRC patient in the training and validation cohorts (including the testing cohort, entire cohort, E-MTAB-1980 cohort, and HSP cohort) received an individual risk score according to this equation. The subjects were subsequently assigned into high- and low-risk groups using the median cutoff risk score as a threshold. Subsequently, Kaplan-Meier curves and ROC curves were applied to assess the prognostic role of the model. To verify the clinical application value of the constructed model, we analyzed the association between the model-based risk score and clinicopathological features based on the TCGA database. Additionally, survival analysis was performed using different subgroups of patients.

### Protein Network Construction

GeneMANIA (http://genemania.org/), a multifunctional and user-friendly web interface, was utilized for predicting interactions and functions of genes and gene sets (26). In this study, we used this web tool to develop a 6-PRG-involved network and to screen other potential binding partners in the regulatory network.

### Evaluation of the Immune Status, Immune Cell Infiltration Fractions, and ICIs Between the Low- and High-Risk Groups

To investigate the immune status of the different groups, we first quantified the enrichment levels of the 29 immune markers in each sample by ssGSEA. The estimated score, stromal score, immune score, and corresponding tumor purity for each patient were subsequently calculated using the ESTIMATE algorithm (27). The expression of HLA-genes was also analyzed. Next, we estimated the relative abundance of LM22 for each contained sample based on gene expression data through CIBERSORT (6). Patients with a *P* value < 0.05 were included, and significance was assessed based on 1,000 permutations. The proportion of immune cells was depicted in the violin map to compare the distributions of LM22 between the subtypes grouped by clustering analysis. To understand the association between the model and tumor immune microenvironment, the expression levels of 17 ICIs were analyzed between the low- and high-risk groups (28).

### Somatic Mutation Analysis

Somatic mutation information of KIRC was downloaded from the TCGA database. The data which included somatic variants were extract from Mutation Annotation Format (MAF) form, and then analyzed by using “maftools” package (29). The waterfall was used to present the mutation landscapes in patients with high- and low-risk groups in the KIRC patients. In this study, the TMB score of each sample was calculated as the number of mutations/length of exons (30Mb). All KIRC samples with somatic mutations were divided into the high- and the low-TMB groups according the median data. Kaplan-Meier analysis was performed to compare the survival difference between low- and high-TMB groups. Moreover, we further assessed the associations of TMB levels with risk score *via* Wilcoxon test.

### TIMER Database and GDSC Database

TIMER (https://cistrome.shinyapps.io/timer/) is a reliable database to analyze the abundance of tumor-infiltrating immune cells (30). The “SCNA” module of the TIMER database was employed to explore the SCNA of risk model-related genes and effect on the infiltration levels of six immune cells.

GDSC (https://www.cancerrxgene.org/) is a public online database for information on drug sensitivity in cancer cells and molecular markers of drug response, providing a unique resource to facilitate the discovery of novel targets for cancer therapies (31). We used GDSC to explore the sensitivity to anticancer drugs associated with the selected risk signature genes.

### Patients and Specimens

From January 2012 and May 2019, 186 KIRC tissue samples were collected from patients at SPH. No patients received chemotherapy or radiotherapy before surgery. The pathological diagnosis was confirmed by two independent pathologists after surgery. All patients were informed of the importance of follow-up and were regularly followed every three months after surgery. All samples were subjected to quantitative real-time polymerase chain reaction (qRT-PCR) analysis. The study was approved by the Ethics Committee of SPH, and all patients signed the informed consents for using their pathological tissues and related information.

### RNA Extraction and qRT-PCR

Total RNA from 186 fresh-frozen KIRC tissue samples was extracted using the RNAiso plus kit (TAKARA) according to the manufacturer’s instructions, and the expression of the model-related genes was further examined by qRT-PCR. The complementary DNA (cDNA) was synthesized with PrimeScript RT Reagent kit (TAKARA) according to the manufacturer’s instructions. The qRT-PCR was performed on LightCycler 480 II System (Roche) using an SYBR Green Master Kit (Roche). Human β-actin was introduced as an internal reference gene to normalize mRNA levels. Expression levels of each mRNA were calculated using the −△Ct method. All trials were conducted in triplicate. The primers are presented in **Supplementary Table 1**.

### Statistical Analysis

The Mann-Whitney U test was used to compare gene expression between tumor tissues and adjacent nontumorous tissues. The Wilcoxon test was used to compare two groups, and the Kruskal-Wallis test was used to compare more than two groups. Chi-squared tests were performed to compare the categorical variables. Qualitative variables were compared using Pearson’s test, where appropriate. Kaplan-Meier analysis was used to evaluate OS, and the log-rank test was used to compare the OS between groups. Univariate and multivariate Cox regression analyses were implemented to identify independent predictors of OS. All statistical analyses were conducted using R version 4.01 and SPSS 24.0 (IBM, NY, USA). If not specified above, *P* < 0.05 was considered statistically significant.

## Results

### The Expression Level of PRGs Is Upregulated in KIRC

**Figure 1:**
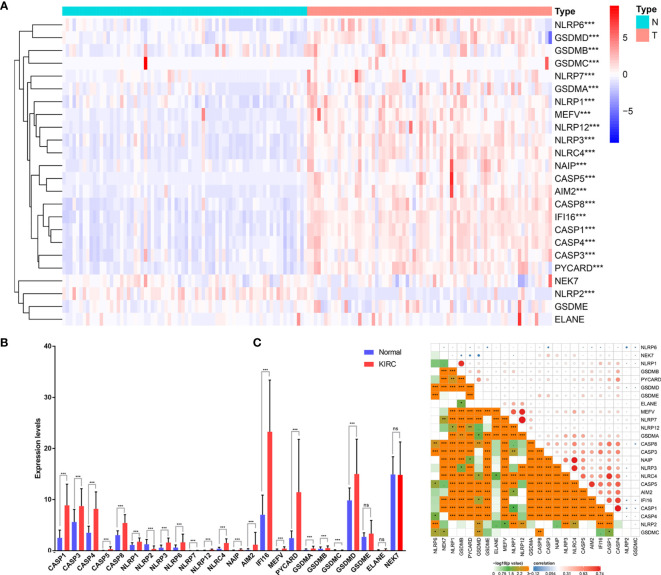
Expression of PRGs in KIRC tissues compared with normal kidney tissues and their interactions. **(A)** Heatmap of the expression of the 24 PRGs in the tumors and normal tissues of the TCGA dataset. **(B)** The expression of PRGs was significantly increased in 72 KIRC compared with that in normal kidney pairs. **(C)** Interaction analysis among the 24 PRGs. *P < 0.05, **P < 0.01, ***P < 0.001. ns, no significance.

To explore the biological functions of PRGs and their significance in KIRC, we initially measured the expression patterns of 24 PRGs in 72 pairs of KIRC samples and adjacent non‐tumor samples based on The Cancer Genome Atlas (TCGA) database. Differential analysis revealed that the expression levels of PRGs between KIRC and normal samples were distinct (**Figures 1A, B**). Twenty-one genes were identified as differentially expressed PRGs, including 20 downregulated genes (NLRP6, GSDMD, GSDMB, GSDMC, NLRP7, GSDMA, NLRP1, MEFV, NLRP12, NLRP3, NLRC4, NAIP, CASP5, AIM2, CASP8, IFI16, CASP1, CASP4, CASP3, and PYCARD) and 1 downregulated gene (NLRP2) in KIRC compared with normal adjacent tissues (*P* < 0.001). Additionally, no significant difference was found in the expression of NEK7, GSDME, and ELANE between KIRC and normal tissues (*P* > 0.05). Collectively, these findings suggest that pyroptosis plays an important biological role during tumorigenesis and disease progression.

To further explore the nature of the interactions among PRGs, we examined the correlation among 24 PRGs. Most of the interactions exhibited a significantly positive correlation between two quantities (**Figure 1C**). Additionally, NLRP7 was most correlated with NLRP12 among all the interactions of 24 PRGs.

### Two Subgroups Are Different in Clinicopathological Features and Survival in KIRC by Consensus Clustering of PRGs

**Figure 2:**
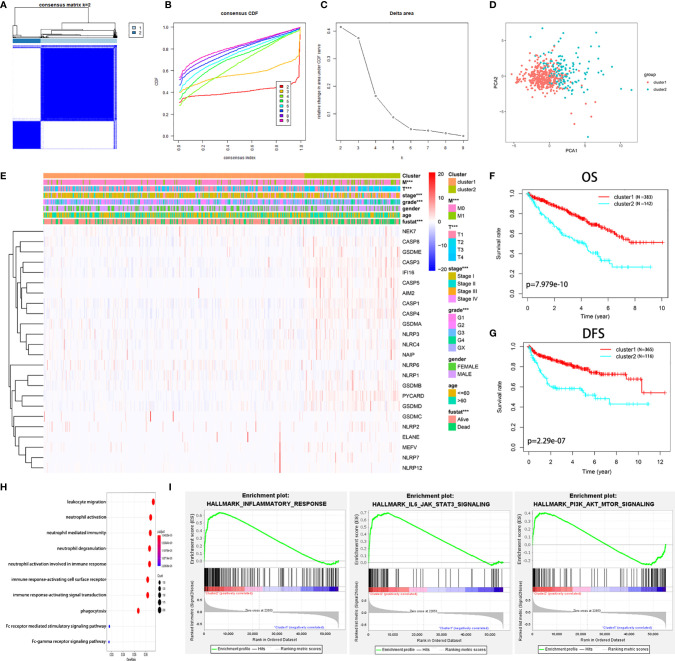
Diverse clinical characteristics and survival of KIRC between cluster 1 and cluster 2 subtypes in the TCGA cohort. **(A)** The TCGA KIRC cohort was divided into two distinct clusters when k = 2. **(B)** Consensus clustering cumulative distribution function (CDF) for k = 2 to 9. **(C)** Relative change in the area under the CDF curve for k = 2 to 9. **(D)** PCA of the TCGA dataset based on the expression profiles of the 24 PRGs. **(E)** Heatmap and distribution of clinicopathological variables between the two clusters. **(F, G)** Kaplan-Meier curves of OS **(F)** and DFS **(G)** for patients with KIRC between the two clusters. **(H)** Biological processes of the genes with different expression between the two clusters. **(I)** GSEA showed that the inflammatory response, IL6-JAK-STAT3 signaling, and PI3K-AKT-mTOR signaling were significantly enriched in cluster 2. ***P < 0.001.

We found that the K-means clustering algorithm with 2 clusters achieved the clearest population clusters and was considered the optimal value. According to the expression levels of the PRGs from the TCGA database, the KIRC samples were clustered into 2 subtypes (cluster 1, n = 383 and cluster 2, n = 142) (**Figures 2A–C**). We then employed principal component analysis (PCA) to study the gene expression pattern between the two subtypes and observed that the distribution pattern of gene expression profiles within the two groups differed (**Figure 2D**). Next, the relationships between the clustering and clinicopathological features were evaluated (**Figure 2E**). Cluster 2 was preferentially associated with a higher M stage (*P* < 0.01), T stage (*P* < 0.01), AJCC stage (*P* < 0.001), and grade (*P* < 0.001), while no significant difference was observed for other parameters, such as age and gender. Additionally, we noticed that cluster 2 showed a shorter overall survival (OS; *P* = 7.979e-10) and disease-free survival (DFS; *P* = 2.29e-07) than cluster 1 (**Figures 2F, G**).

The genes that were significantly altered between the two groups were subjected to gene ontology (GO) analysis. The results were closely related to immune-related biological processes, including leukocyte migration, neutrophil activation, and neutrophil-mediated immunity (**Figure 2H**). Subsequently, gene set enrichment analysis (GSEA) was conducted, indicating that immune- and cancer-related hallmarks, including the inflammatory response, IL6-JAK-STAT3 signaling, and epithelial-mesenchymal transitions signaling, had significant correlations with cluster 2 (**Figure 2I**). The above results demonstrated that the two subgroups determined based on the expression of the PRGs were strongly linked to the malignancy of KIRC.

### Construction of the Prognostic Risk Model Based on the TCGA Training Cohort

**Figure 3:**
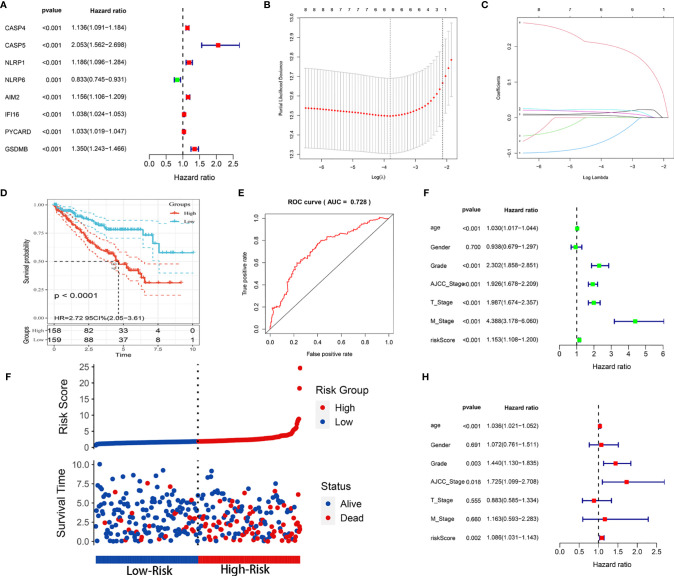
Construction of the prognostic risk model based on the TCGA training cohort. **(A)** Forest map of 8 PRGs significantly correlated with OS and identified by Cox univariate analysis. **(B)** Screening of optimal parameters (lambda) in the LASSO regression model based on the TCGA training cohort. **(C)** LASSO coefficient profiles of the 8 PRGs determined by the optimal lambda. **(D)** Kaplan-Meier curve for the OS of KIRC patients in the high- and low-risk groups in the TCGA training cohort. **(E)** ROC analysis of the prognostic model regarding the OS and survival status in the TCGA training cohort. **(F)** Scatterplots in the top and bottom panels illustrate the distribution of the risk score and survival status in the TCGA training patients, respectively. **(G, H)** Univariate **(G)** and multivariate **(H)** Cox regression analyses of the risk score and clinicopathological parameters in the TCGA training cohort.

Because we identified distinct expression patterns in KIRC patients, we next considered that constructing a pyroptosis-related risk signature might be useful for predicting prognosis. We first conducted a univariate Cox regression analysis and identified 8 PRGs (CASP4, CASP5, NLRP1, NLRP6, AIM2, IFI16, PYCARD, GSDMB) that were correlated with OS in the training cohort (*P* < 0.05) (**Figure 3A**). All eight PRGs, except NLRP6, were considered risk genes with HRs > 1. Based on the above results, to further clarify the prognostic potential, we subsequently conducted LASSO analysis on the expression values of 8 prognostic PRGs (**Figures 3B, C**). Ultimately, 6 genes, CASP4, NLRP6, AIM2, IFI16, PYCARD, and GSDMB, were identified to construct the prediction model. The prognostic risk model was established based on the following formula: risk score = (0.0137 × expression value of CASP4) – (0.0624 × expression value of NLRP6) + (0.0227 × expression value of AIM2) + (0.0149 × expression value of IFI16) + (0.0059 × expression value of PYCARD) + (0.2049 × expression value of GSDMB). The risk score for each patient in the TCGA training cohort was calculated, and the patients were stratified into a high-risk group and a low-risk group according to the median risk score. Kaplan-Meier analysis showed that the prognosis of the KIRC patients in the high-risk group was poorer than that in the low-risk group (*P* < 0.0001; **Figure 3D**). The prognostic model showed a satisfactory prediction efficiency, with an area under the ROC curve (AUC) value of 0.728 (**Figure 3E**). Additionally, the risk score distributions and patient survival status are shown in **Figure 3F**.

Univariable and multivariable Cox regression analyses were utilized to identify whether the model-based risk score could be an independent predictor of OS. The results showed that age, grade, AJCC stage, T stage, M stage, and risk score were closely related to OS (*P* < 0.001) in univariate analysis (**Figure 3G**). Likewise, age (*P* < 0.001), grade (*P* = 0.003), AJCC stage (*P* = 0.018), and risk score (*P* = 0.002) maintained their prognostic values in multivariate Cox analysis (**Figure 3H**). Therefore, these data demonstrated that the risk score was an independent prognostic indicator for patients with KIRC.

### Internal and External Validation of the Prognostic Risk Model in KIRC Patients

**Figure 4:**
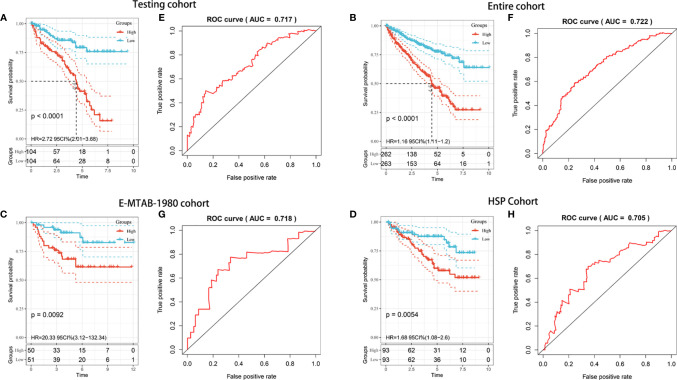
Internal and external validation of the prognostic risk model in KIRC patients. **(A–D)** Kaplan–Meier survival analysis of OS between patients with low-risk scores and high-risk scores in the TCGA testing cohort **(A)**, TCGA entire cohort **(B)**, E-MTAB-1980 cohort **(C)**, and HSP cohort **(D)**. **(E–H)** ROC analysis of the prognostic model in the TCGA testing cohort **(E)**, TCGA entire cohort **(F)**, E-MTAB-1980 cohort **(G)**, and HSP cohort **(H)**.

To explore whether the prognostic model was generalizable and harbored similar prognostic value in different populations, we applied it to the internal (TCGA testing and entire) and independent external (E-MTAB-1980 and HSP) validation cohorts. Regarding the predictions in the TCGA testing cohort, Kaplan-Meier analysis showed that patients with high risk scores had worse OS (*P* < 0.001) (**Figure 4A**). The AUC value for predicting OS in the TCGA testing cohort was 0.717 (**Figure 4E**). For the TCGA entire cohort, the model could still separate analytic samples into various subgroups of clinical importance. The Kaplan-Meier survival curve indicated that patients in the high-risk group exhibited a significantly lower OS than those in the low-risk group (*P* < 0.001) (**Figure 4B**). The AUC value of the entire TCGA cohort was 0.772, which was comparable to the model results described above (**Figure 4F**). Next, External validation using the E-MTAB-1980 and HSP cohorts was performed to validate the robustness and validity of the constructed model. Consistent with TCGA analysis, Kaplan-Meier analysis suggested that the patients in the high-risk group had a significantly shorter OS within both the E-MTAB-1980 cohort and HSP cohort **(** **Figures 4C, D** **).** The AUC values of the E-MTAB-1980 cohort and HSP cohort were found to be 0.711 and 0.705, respectively (**Figures 4G, H**). The risk score distributions and patient survival status in four cohorts were shown in **Supplementary Figure 1**. Overall, the risk score showed favorable discrimination ability in all four cohorts.

### Clinical Evaluation of the Prognostic Risk Model Based on the TCGA Entire Cohort

**Figure 5:**
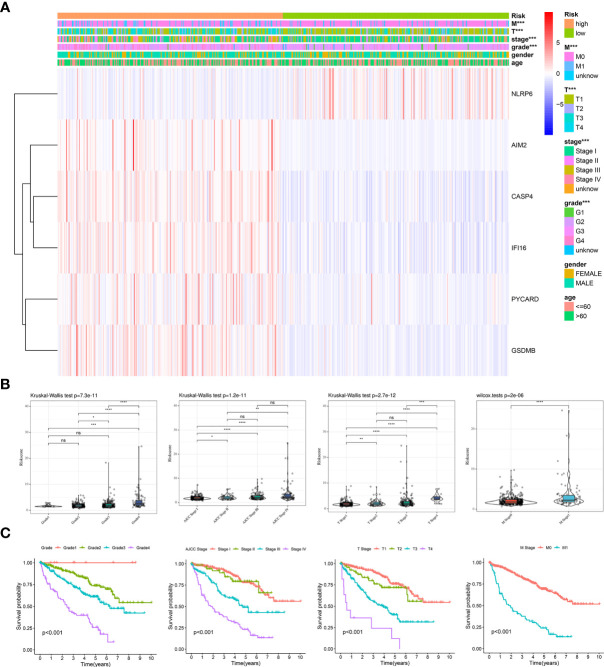
Clinical evaluation of the prognosis risk model based on the TCGA entire cohort. **(A)** Heatmap of the expression of 6 PRGs and distribution of clinical features between the low- and high-risk groups. **(B, C)** Expression of the model-based risk score **(B)** and Kaplan-Meier survival analysis **(C)** in KIRC patients stratified by different clinicopathological characteristics (grade, AJCC stage, T stage, and M stage). *P < 0.05, **P < 0.01, ***P < 0.001, ****P < 0.0001. ns, no significance.

To validate the clinical value of the prognostic model, we evaluated the relationship between the risk score and clinical features. A heatmap was used to visualize differences in the expression levels of the six genes between the low- and high-risk groups. The analysis demonstrated that risk genes (CASP4, AIM2, IFI16, PYCARD, GSDMB) were upregulated in the high-risk group, while the expression of protective genes (NLRP6) was downregulated (**Figure 5A**). Additionally, a significant difference was found among the diverse groups in terms of the M stage (*P* < 0.001), T stage (*P* < 0.001), AJCC stage (*P* < 0.001), and grade (*P* < 0.001). We also noticed that the risk score increased with the progression or severity of the tumor (**Figure 5B**).

Subsequently, stratified survival analyses were performed to examine the good applicability of our prognostic model. As expected, the patients with Grade 1 disease showed the best prognosis, followed by those with Grade 2, Grade 3, and Grade 4 disease. Furthermore, similar trends were presented in the AJCC stage, T stage, and M stage (**Figure 5C**). We next conducted stratified survival analyses according to the different clinical features. Excitingly, we observed that the patients with high-risk scores were associated with a shorter OS across all the subgroups (**Supplementary Figure 2**). Thus, the dysregulation of pyroptosis is critically involved in the development and progression of KIRC.

### Analysis of Network and Gene Set Enrichment Analysis (GSEA)

**Figure 6:**
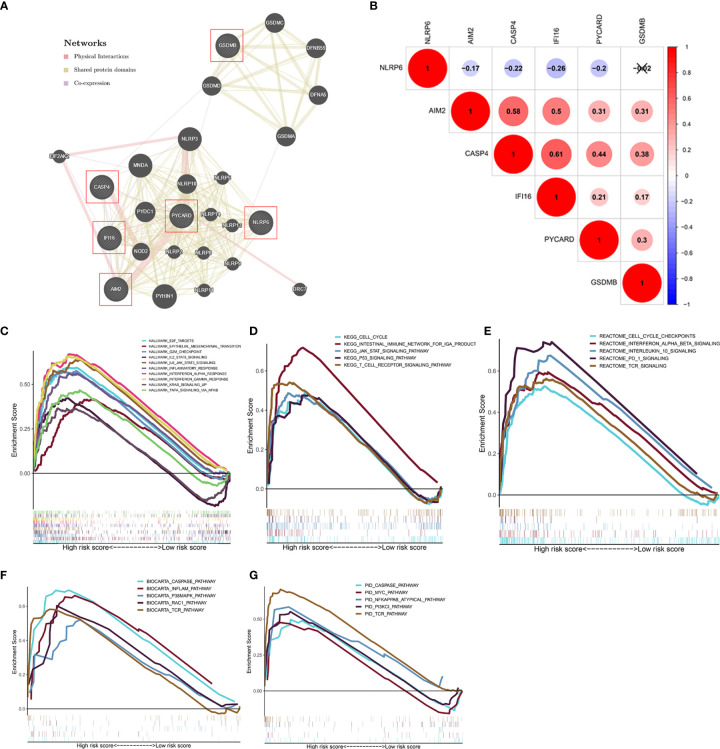
Analysis of the regulatory network and gene sets associated with high-risk groups. **(A)** The regulatory network involving six model-related genes and twenty potential binding proteins was constructed through GeneMANIA. **(B)** Correlation analysis of the six genes. **(C–G)** GSEA showed the significantly enriched Hallmark **(C)**, KEGG **(D)**, Reactome **(E)**, BioCarta **(F)**, and PID **(G)** gene sets in high-risk score based on the TCGA database.

A gene interaction network was visualized using GeneMANIA to gain further insight into the possible relationships between the six PFRs and their potential binding partners. The regulatory network carried twenty-six genes, including six target PFRs and additional twenty genes that were recognized automatically through GeneMANIA (**Figure 6A**). We then analyzed the correlation of the six genes in KIRC and found that the interaction between CASP4 and IFI16 (r = 0.61) was most significant and displayed a positive correlation (**Figure 6B**).

GSEA was performed to investigate the relevant biological processes and signaling pathways using the pyroptosis model based risk score for classification. The results suggested that cancer- and immune-related ‘Hallmark’ gene sets, such as epithelial-mesenchymal transition, inflammatory response, PI3K/AKT/mTOR signaling pathway, and Wnt/β-catenin signaling pathway that were highly enriched in the high−risk phenotype (**Figure 6C**). Moreover, several classical pathways from KEGG, Reactome, BioCarta, PID gene sets, including the cell cycle, caspase pathway, Myc pathway were also related to the high−risk group (**Figures 6D–G**).

### Prognostic Risk Scores Related to Different Immune Statuses, Immune Cell Infiltration and ICIs

**Figure 7:**
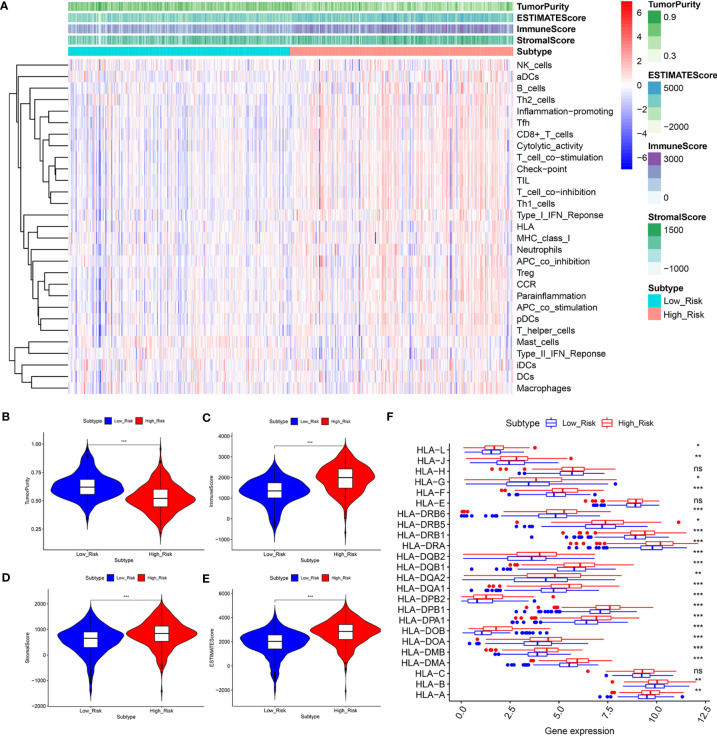
The low‐ and high‐risk groups display different immune statuses. **(A)** Heatmap of the distribution of 29 immune-related genes between the low‐and high‐risk groups using ssGSEA. **(B–F)** Expression level of the tumor purity **(B)**, ESTIMATE score **(C)**, immune score **(D)**, stromal score **(E)**, and HLA-related genes between the low‐ and high‐risk groups. *P < 0.05, **P < 0.01, ***P < 0.001. ns, no significance.

According to the results shown above, to further assess the relationship between immune status between the groups, the relative quantities of 29 immune markers were systematically evaluated using single-sample GSEA (ssGSEA). A heatmap was constructed to depict a more comprehensive immune infiltration landscape for the TCGA KIRC cohort (**Figure 7A**). We used the ESTIMATE algorithm to successfully generate the tumor purity score, estimate score, immune score, and stromal score. Notably, patients with a low-risk score presented a higher level of tumor purity (*P* < 0.001) and a lower estimate score (p < 0.001), immune score (*P* < 0.001), and stromal score (*P* < 0.001) than those with a high-risk score (*P* < 0.001) (**Figures 7B–E**), consistent with previous study findings that a lower estimate score represents higher tumor purity. Considering that human leukocyte antigen (HLA)-related genes play an essential role in regulating the immune response, we then compared the expression of HLA-related genes between different groups and found that most of the HLA-related genes were upregulated in the high-risk group (**Figure 7F**).

**Figure 8:**
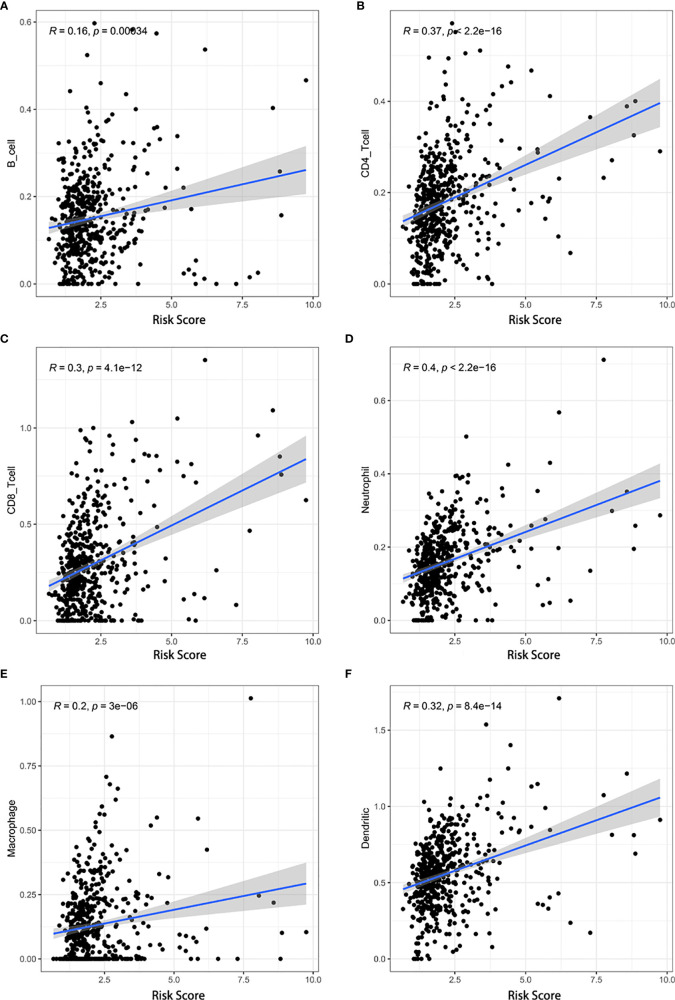
Associations between the risk score and infiltration levels of six immune cell types. **(A)** B cells, **(B)** CD4+ T cells, **(C)** CD8+ T cells, **(D)** neutrophils, **(E)** macrophages, and **(F)** dendritic cells.

Additionally, we analyzed the relationship between the risk score and infiltration levels of six immune cell types (B cells, CD4+ T cells, CD8+ T cells, neutrophils, macrophages, and dendrites). Interestingly, a significantly positive correlation was found between the risk score and content of the six immune cell types (**Figure 8**). The pyroptosis-related risk model effectively reflected the status of the immune microenvironment for KIRC patients.

Subsequently, we estimated the fraction of 22 tumor-infiltrating immune cells (LM22) in the low- and high-risk groups using CIBERSORT. The bar plot illustrates the specific fractions of LM22 in each KIRC sample (**Supplementary Figure 3A**). Additionally, we depicted the distributions of LM22 between the two groups in the heatmap (**Supplementary Figure 3B**). We observed a dependency among the various immunocyte subpopulation fractions (**Supplementary Figure 3C**). Finally, we compared the differential infiltration of 22 immune cells between the groups. The low-risk group had higher infiltration levels of resting CD4 memory T cells, gamma delta T cells, monocytes, M2 macrophages, resting dendritic cells, activated dendritic cells, resting mast cells, and eosinophils, whereas infiltration was more correlated with plasma cells, CD8 T cells, activated CD4 memory T cells, follicular helper T cells, and regulatory T cells (Tregs) (**Supplementary Figure 3D**).

Recent breakthroughs in tumor immunology have generated substantial interest in the potential of ICIs to treat other solid tumors. To further understand the relationship between the model and ICIs, 17 ICIs (B7-H3, B7-H4, CTLA4, CD27, ICOS, TIGIT, PD-1, LAG3, CD58, CD86, PD-L1, PD-L2, TIM-3, CD270, CD70, CD40, and IDO1) were analyzed as reported previously. We discovered that high risk scores were positively correlated with high expression of ICIs, in addition to B7-H4, PD-L1, and CD40 (**Supplementary Figure 4**).

### Tumor Somatic Mutational Landscape and Effect of Genetic Mutants of Model-Related PRGs on Immune Cell Infiltration

**Figure 9:**
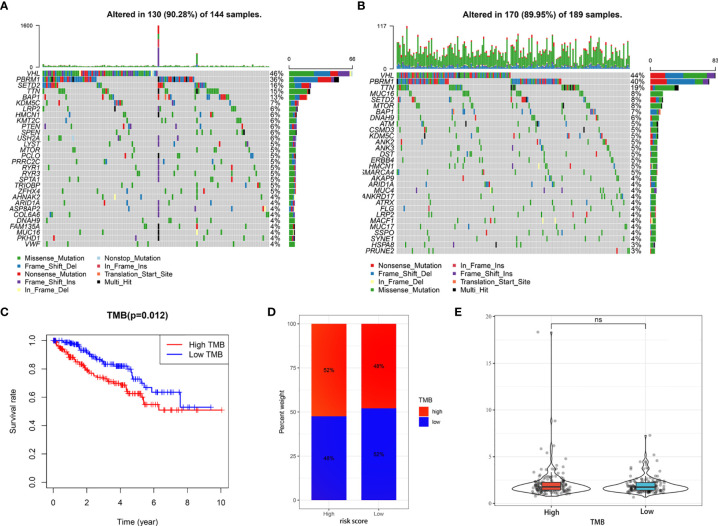
Tumor somatic mutational analyses between high- and low-risk scores. **(A, B)** Waterfall plot shows the mutation distribution of the top 30 most frequently mutated genes in the high-risk group **(A)** and low-risk group **(B)**. **(C)** Survival analysis of OS in KIRC patients between high- and low-TMB groups. **(D)** Difference in TMB between the high- and low-risk groups. **(E)** Difference in risk scores between the high- and low- TMB groups. ns, no significance.

Giving that gene mutations are an important cause of tumorigenesis, we explored the differences in the distribution of somatic mutations between high- and low-risk groups. The top 30 most frequently mutated genes of these two groups were displayed in **Figures 9A, B**, respectively. The Kaplan-Meier curves for OS indicated that the patients with high-TMB group had significantly worse OS than those with low-TMB group (**Figure 9C**). In addition, the high-risk group presented more extensive TMB than the low-risk group (**Figure 9D**). Interestingly, however, there was no statistical difference in the expression level of risk score between the low- and high-TMB groups (**Figure 9E**).

**Figure 10:**
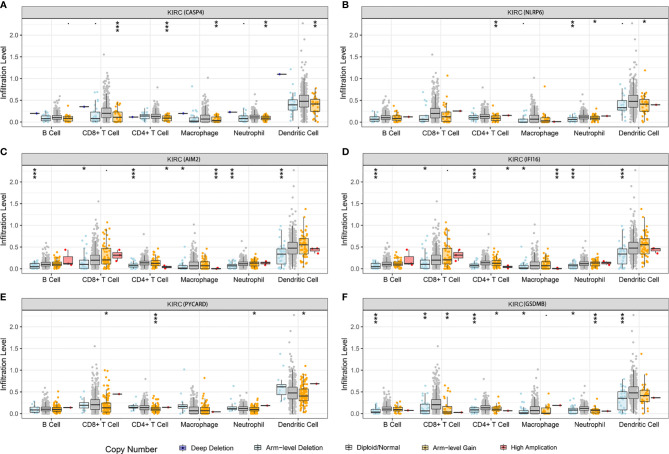
Relationship between the mutants of six model-related PRGs and immune cell infiltration. **(A)** CASP4, **(B)** NLRP6, **(C)** AIM2, **(D)** IFI16, **(E)** PYCARD, and **(F)** GSDMB. *P < 0.05, **P < 0.01, ***P < 0.001.

**Figure 11:**
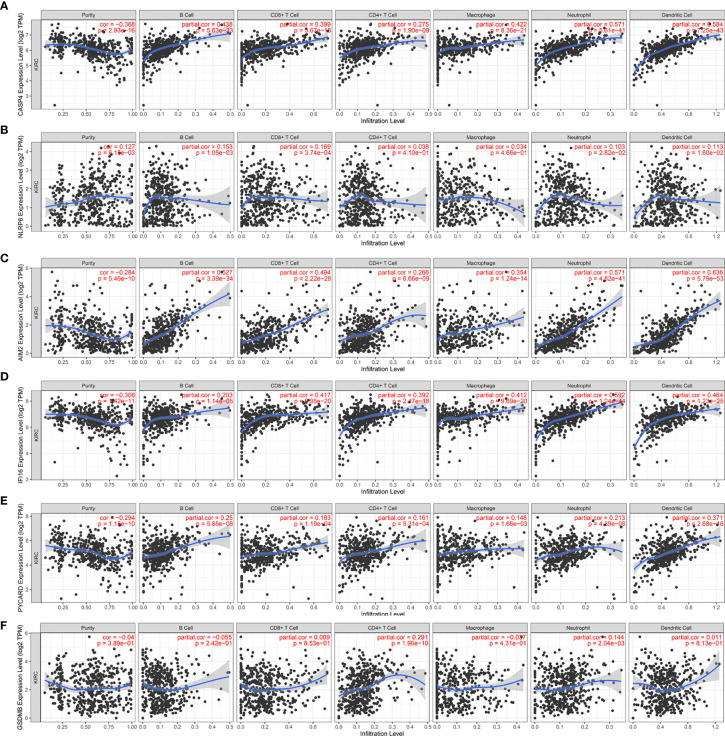
Correlation of six model-related PRGs expressions with immune infiltration levels in KIRC. **(A)** CASP4, **(B)** NLRP6, **(C)** AIM2, **(D)** IFI16, **(E)** PYCARD, and **(F)** GSDMB.

We further investigated the underlying relationships between the somatic cell copy number alternations (CNAs) of these model-related genes and different immune cell infiltrations using the Tumor Immune Estimation Resource (TIMER) database. The mutants of these six genes were strongly associated with the immune infiltration microenvironment in KIRC. Compared with the immune infiltration levels in samples with wild-type genes, diverse forms of mutations carried by these six genes displayed lower levels of immune infiltrates. Among the CNAs of the six identified model-related genes, arm-level deletion and arm-level gain exhibited a statistically significant effect on the immune cell infiltration levels in KIRC (**Figure 10**). In addition, to further understand the relationship between six model-related genes and immune infiltration in KIRC microenvironment, we explored the correlation ship in TIMER. The results illuminated that the expression of these genes were positively correlated with the infiltrating levels of immune cells (**Figure 11**).

### Drug Sensitivity Analysis of Model-Related PRGs

**Figure 12:**
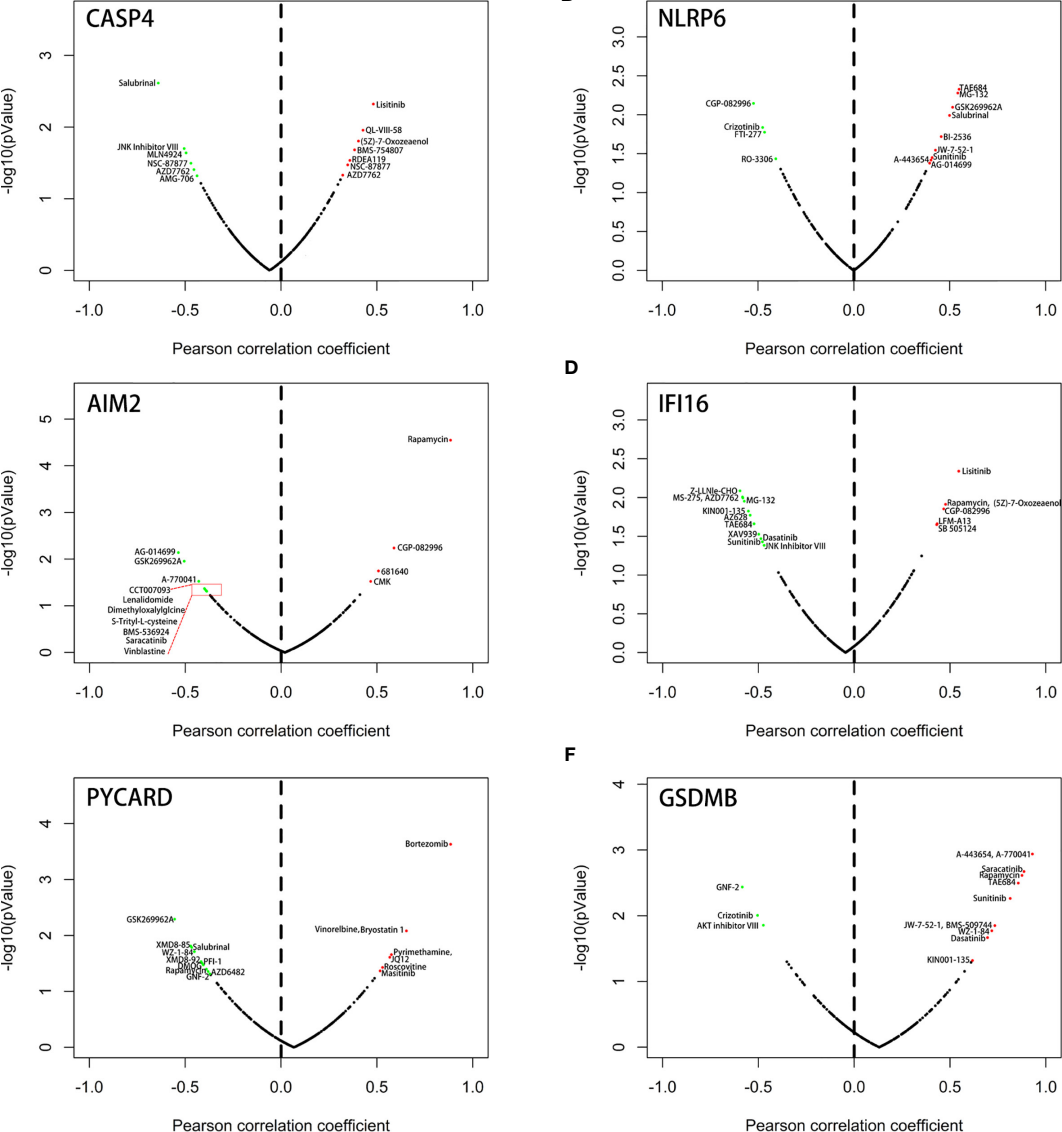
Correlation between the expression status of 6 six model-related PRGs and drug sensitivity of KIRC cell lines. **(A)** CASP4, **(B)** NLRP6, **(C)** AIM2, **(D)** IFI16, **(E)** PYCARD, and **(F)** GSDMB.

We next used the Genomics of Drugs Sensitivity in Cancer (GDSC) database to identify an association between sensitivity to anticancer drugs and the expression levels of the six genes. The results indicated that the six genes were frequently associated with the resistance or sensitivity of kidney cancer cells to multiple targeted drugs (**Figure 12**). Among these six genes, NLRP6, IFI16, and GSDM8 were relatively important because their expression levels were closely associated with sunitinib. Moreover, the expression of NLRP6 and GSDM8 was negatively correlated with sunitinib resistance. However, the expression of IFI16 was positively correlated with sunitinib resistance.

## Discussion

Pyroptosis is a highly inflammatory form of programmed cell death that is characterized by inflammasome activation and the secretion of IL-1β and IL-18 (32, 33). Dysregulation of pyroptosis may cause dysfunction in the stimulation of adaptive immune defenses and contribute to the initiation and progression of multiple tumors (17, 34). However, controversies exist concerning the role of PRGs as tumor suppressors or tumor promoters. For example, Wang et al. (20) reported that GSDMD was downregulated in gastric cancer and exerted a tumor suppressor role by inhibiting the PI3K/AKT signaling pathway. Conversely, Gao et al. (35) found that GSDMD protein was significantly upregulated and promoted cell proliferation and a poor prognosis by potentiating the EGFR/AKT signaling pathway in lung cancer. The distinct effect of PRGs in different tumor cells reflects the overwhelmingly complex molecular regulation mechanism of pyroptosis. Because most of the studies primarily concentrated on the intrinsic oncogenic pathways of malignant tumors, it is indispensable to elucidate the potential regulatory mechanisms of pyroptosis that may significantly affect the characteristics of the cancer treatment response, particularly precision immunotherapy. Furthermore, the detailed effects of pyroptosis on the TME of KIRC remain to be fully investigated.

In this study, we sought to explore the expression patterns of pyroptosis in KIRC and its prognostic value and effect on the TME. The expression of NLRP2 was significantly decreased in KIRC tissues compared with that in normal tissues, whereas NEK7, GSDME, and ELANE were not significantly different. The expression levels of other PRGs were higher in KIRC tissues than in noncancerous tissues. Next, we then determined two subtypes of KIRC—namely, cluster 1 and cluster 2—by consensus clustering based on the expression profiles of PRGs from the TCGA database. The diverse subtypes affected the prognosis and showed significant differences in clinicopathological features and tumor immune infiltrations. The patients in cluster 2 were found to be closely related to a more advanced tumor stage and grade. As predicted, cluster 1 presented better OS and DFS than cluster 2. GO enrichment analysis and GSEA were conducted to further explore the functions associated with different subgroups. Several biological processes correlated with immunity were identified, including leukocyte migration, neutrophil activation, and neutrophil-mediated immunity. A previous study suggested that leukocyte migration might contribute to the pathogenesis of many human diseases, including tumors (36). Additionally, increasing evidence has revealed that the immune system is involved in carcinogenesis and tumor progression by promoting cancer cell proliferation, migration, immune escape and chemotherapy resistance (37). GSEA revealed that the characteristic features of malignant tumors, including IL6-JAK-STAT3 signaling and PI3K/AKT/mTOR signaling, were obviously related to cluster 2. Wang et al. found that the downregulation of GSDMD markedly promoted the proliferation of gastric cancer through inactivating the STAT3 and PI3K/AKT pathways (20). Similarly, Chen et al. found that downregulated AIM2 expression may be involved in the PI3K/AKT signaling pathway in colorectal cancer (38). Here, we suggest that pyroptosis is related to many biological processes and signaling pathways, revealing their significant roles in the initiation and development of KIRC.

We then constructed a prognostic prediction model in the training cohort. The risk scoring system based on six genes predicted the prognosis of KIRC patients, and the patients were effectively stratified into high- and low-risk groups. Patients in the high-risk group had a significantly shorter OS than those in the low-risk group. The performance of the prognostic pyroptosis-relevant model was confirmed in two internal cohorts. The independent external E-MTAB-1980 and HSP cohorts also yielded consistent results. Additionally, the risk score increased with the progression or severity of the tumor. Univariate and multivariate Cox analyses indicated that the six-gene prognosis model is an independent factor. Among these six model-related PRGs, the expression of NLRP6 was significantly decreased in high-risk KIRC patients. Surprisingly, NLRP6 was upregulated in normal tissue samples compared with that in KIRC tissue, likely because of the different effects of NLRP6 at different stages in KIRC tumorigenesis and development. Chen et al. suggested that NLRP6 plays a fundamental role in maintaining intestinal homeostasis, thus preventing intestinal tissue from aberrant inflammation and tumors (39). AIM2 has been identified as a tumor-suppressive gene in human colorectal cancer (38), but Zhang et al. (40) showed that AIM2 promotes non-small cell lung cancer progression through an inflammasome-dependent pathway. One previous study found that caspase-4 is highly expressed in the lamina propria of colorectal cancer compared with that in normal tissues, indicating that caspase-4 may represent a biomarker of colon carcinoma (41). IFI16 and PYCAED serve as oncogenes in cervical cancer and gastric cancer, respectively (42, 43). Accumulated evidence indicates that GSDMB is overexpressed in several cancer types and may be involved in cancer progression and metastasis (44). These studies revealed that the dysregulation of pyroptosis might play divergent roles in different types of cancer.

The tumor microenvironment plays a critical regulatory role in carcinogenesis and tumor progression (45). According to our scoring system, the difference in the TME between the low-risk and high-risk groups was notable. The immune score and expression levels of HLA-related genes in the high-risk group were significantly higher than those in the low-risk group, while the tumor purity exhibited the opposite trend, likely explaining why the low-risk group patients had a higher survival. Our observation agreed with that reported by Zeng et al. (46), suggesting that the OS of patients with low immune scores is better than that of patients with high immune scores. By contrast, low tumor purity was responsible for glioma’s aggressive phenotype and poor prognosis (47). KIRC is considered an immunogenic tumor; however, to a large extent, it mediates immune dysfunction by inducing immunosuppressive cells to infiltrate the tumor microenvironment (48). Currently, the investigation of PRGs in the TME in KIRC is insufficient. In the present study, the model-based risk score was positively associated with the infiltration of six immune cell types. This finding is consistent with a previous study finding that high-risk glioma patients with higher immune cell infiltration levels show a poorer prognosis (49). These findings indicated that pyroptosis was, in part, involved in the regulation of the TME. Additionally, our research suggested that the CNAs of PRGs might affect the immune cell infiltration levels in KIRC, providing new insights for future TME studies. Taken together, the results show that the prognostic model may serve as an indicator for outcome and immune cell infiltration, holding promising prospects in modern clinical practice.

Presently, numerous clinical trials are underway that evaluate the effect of ICIs in KIRC patients. By exploring the correlation between the risk score and expression of critical ICIs, we further noticed that most ICIs (14/17) presented higher expression in the high-risk group. Based on these observations, we strongly suggest the critical role of the immunosuppressive microenvironment in these patients with a poor prognosis. Hence, patients with high risk scores might benefit most from ICIs compared with patients with low risk scores. We also found that these six model-related genes were associated with targeted therapies. NLRP6, IFI16, and GSDMB were associated with sensitivity to sunitinib. Moreover, some were associated with other targeted therapies, thereby determining a superior agent or treatment strategy for individual patients and expanding insights into future therapeutics for treating KIRC.

Our research had limitations. First, the prospective, larger multicenter trials are required to provide high-level evidence for clinical application. Moreover, the underlying mechanisms of the selected genes in our model should be explored to better study the molecular mechanisms involved in tumorigenesis and the development of KIRC.

In summary, we systematically analyzed the prognostic value, roles in the TME, response to ICIs, and drug sensitivity of PGRs in KIRC. Two KIRC subtypes (clusters 1/2) with diverse outcomes were identified by consensus clustering based on the expression profile of PRGs. The pyroptosis-related prognostic risk model developed from six PRGs can stratify KIRC patients into low- and high-risk subgroups with diverse prognoses and immune cell infiltration. The signature also suggests that the patients with high-risk scores might benefit most from ICIs. Pyroptosis may be involved in targeted therapies for patients with KIRC. Our findings may provide new insight into the role of pyroptosis in the TME in KIRC patients. In conclusion, our prognostic model showed potential clinical usefulness that may improve survival and even develop new therapeutic strategies for KIRC patients.

## Data Availability Statement

Publicly available datasets were analyzed in this study. This data can be found here: TCGA (https://tcga-data.nci.nih.gov/tcga/), ArrayExpress (https://www.ebi.ac.uk/arrayexpress/), TIMER (https://cistrome.shinyapps.io/timer/), and GDSC (https://www.cancerrxgene.org/) websites.

## Ethics Statement

The studies involving human participants were reviewed and approved by the Ethics Committee of Shandong Provincial Hospital (Jinan, China). The patients/participants provided their written informed consent to participate in this study.

## Author Contributions

ZS designed the study and drafted the manuscript. CJ collected and analyzed the data. MZ and ZW prepared tables and figures. XG and FK revised the manuscript. SJ and HW supervised the study. All authors contributed to the article and approved the submitted version.

## Funding

The research was supported by the Shandong key research and development program, China (2019GSF108263).

## Conflict of Interest

The authors declare that the research was conducted in the absence of any commercial or financial relationships that could be construed as a potential conflict of interest.

## Publisher’s Note

All claims expressed in this article are solely those of the authors and do not necessarily represent those of their affiliated organizations, or those of the publisher, the editors and the reviewers. Any product that may be evaluated in this article, or claim that may be made by its manufacturer, is not guaranteed or endorsed by the publisher.
