# Supplementary material for: Comprehensive Analysis of the Immune Infiltrates of Pyroptosis in Kidney Renal Clear Cell Carcinoma
Source: Front Oncol. 2021 Sep 9;11:716854. doi: 10.3389/fonc.2021.716854 (PMC8459616; doi:10.3389/fonc.2021.716854)
Supplement: Supplementary file 1 [file DataSheet_1.docx]

**Supplementary Methods**

**Supplementary Table1. Primer Sequences for qRT-PCR.**

| Gene Name | Forward Primer | Reverse Primer |
| --- | --- | --- |
| CASP4 | 5'-GAAAGAGCTGAAGAGATCTATCCAA-3' | 5'-AGCTCCTTCATCCCTGTGATGT-3' |
| NLRP6 | 5'-GGCAGCAGTTCTCAAGGCA-3' | 5'-ATGGCACAGTGGGTCAGTCA-3' |
| AIM2 | 5'-AGTGGTTTCTTAGAGGTAAATAGCG-3' | 5'-TTCTGATAATGTTCAGCGGGAC-3' |
| IFI16 | 5'-GCTTGAAGACCTGGCTGAAAC-3' | 5'-TTGACAGTGCTGCTTGTGGAG-3' |
| PYCARD | 5'-TACGGGAAGGTCCTGACGG-3' | 5'-TCCGCATCTTGCTTGGGTT-3' |
| GSDMB | 5'-TGGATAATGTAGACTCAACGGGA-3' | 5'-GGTAGCCAGATACTGCTGGGA-3' |
| β-actin | 5′-TTGCGTTACACCCTTTCTTG-3′ | 5′-CACCTTCACCGTTCCAGTTT-3′ |


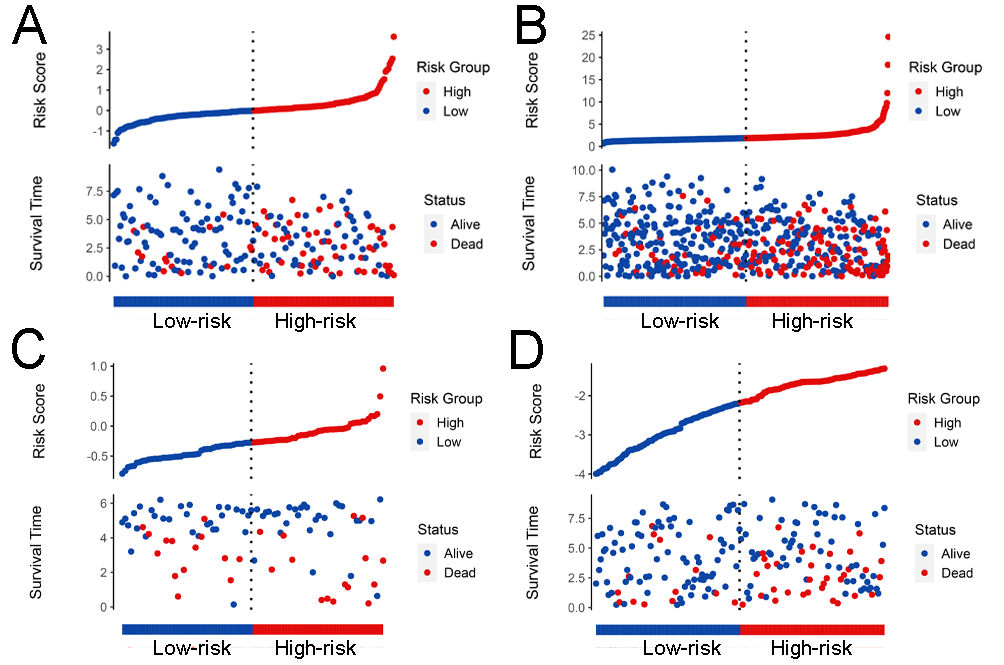


**Supplementary Figure 1. The risk score distributions and patient survival status in four cohorts.** (A) TCGA testing cohort, (B) TCGA entire cohort, (C) E-MTAB-1980 cohort, (D) and HSP cohort.


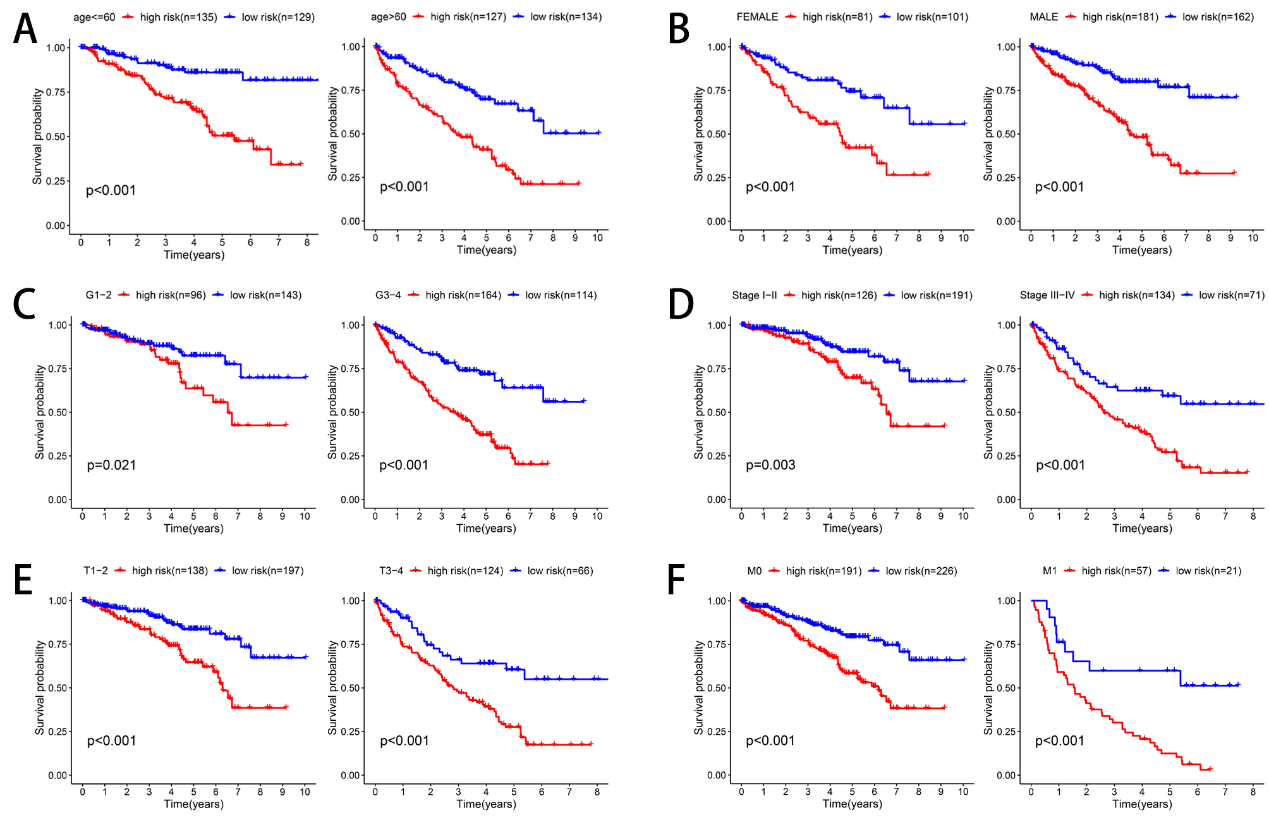


**Supplementary Figure 2. Kaplan‐Meier survival curves in TCGA cohort stratified by different clinical features.** (A) Age (≤60 and >60), (B) Gender (female and male), (C) grade (G1-2 and G3-4), (D) AJCC stage (AJCC stage 1-2 and AJCC stage 3-4), (E) T stage (T1-2 and T3-4), and (F) M stage (M0 and M1).


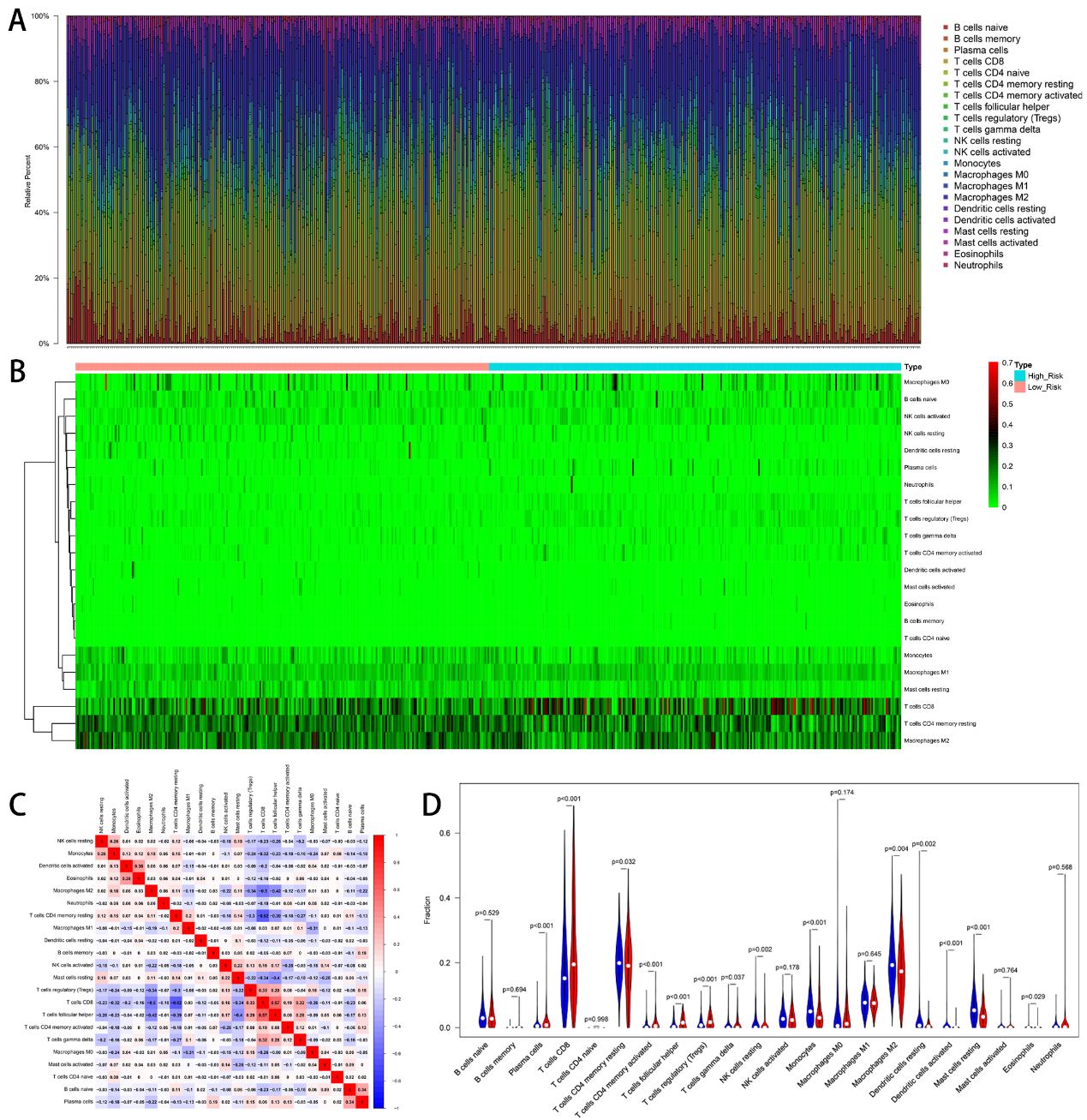


**Supplementary Figure 3. Landscape of immune cell infiltration between the low‐ and high‐risk groups.** (A) The 22 specific immune cell fractions represented by various colors in each sample are shown in a bar plot. (B) Distributions of 22 immune cell fractions between the low- and high-risk groups in the heatmap. (C) Cellular interaction of the 22 tumor-infiltrating immune cell types. (D) Comparison of 22 different immune cell fractions in patients with low- and high-risk scores.


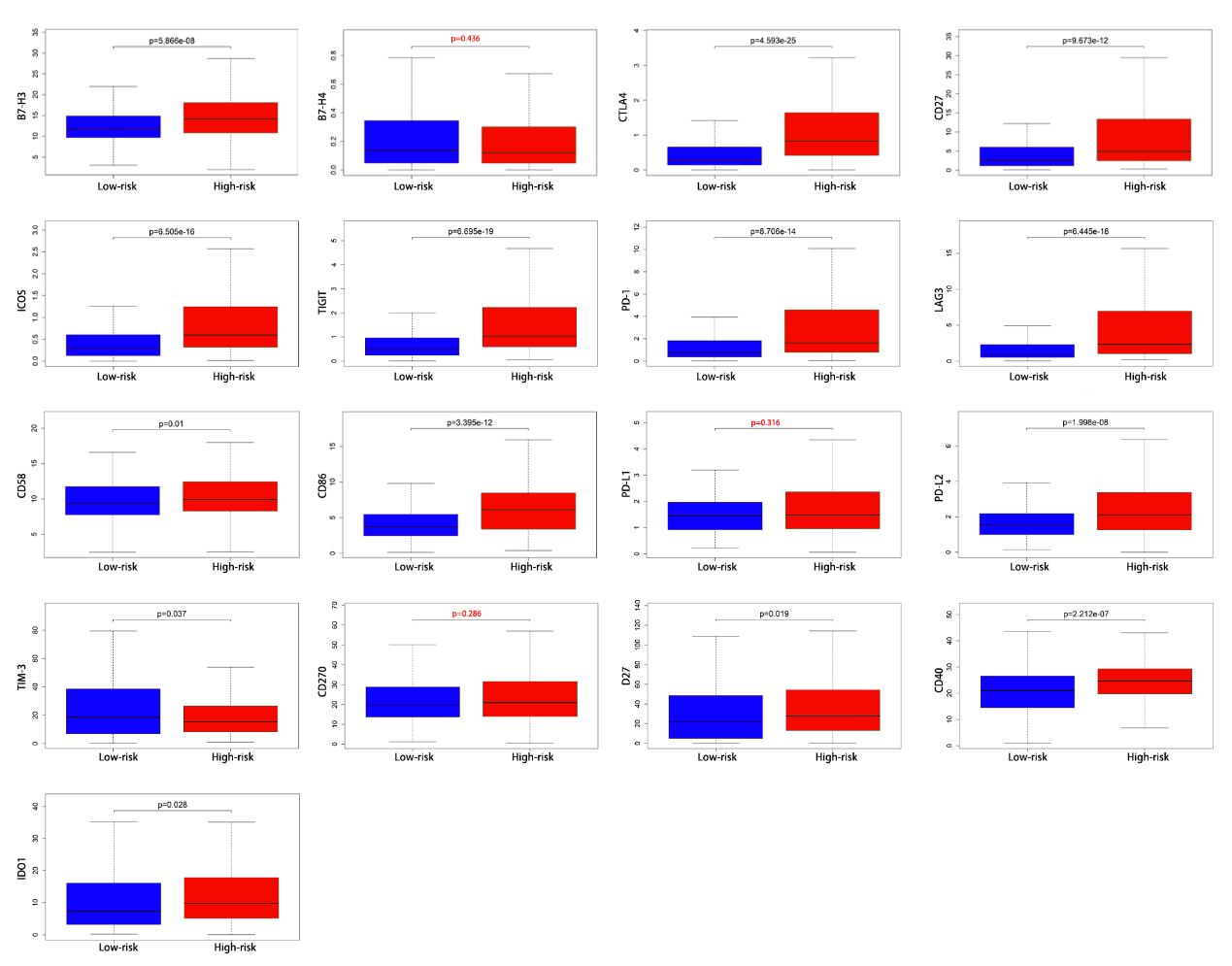


**Supplementary Figure 4. Distribution of 17 ICIs between the low‐ and high‐risk groups.** P values marked in red represent no statistical significance.
